# Learning to interpret topographic maps: Understanding layered spatial information

**DOI:** 10.1186/s41235-016-0002-y

**Published:** 2016-09-22

**Authors:** Kinnari Atit, Steven M. Weisberg, Nora S. Newcombe, Thomas F. Shipley

**Affiliations:** 1 Department of Psychology, Northwestern University, Evanston, IL 60208 USA; 2 Center for Cognitive Neuroscience, University of Pennsylvania, Philadelphia, PA 19104 USA; 3 Department of Psychology, Temple University, Philadelphia, PA 19122 USA

**Keywords:** Topographic maps, Gesture, Speech, Diagrammatic reasoning

## Abstract

Novices struggle to interpret maps that show information about continuous dimensions (typically latitude and longitude) layered with information that is inherently continuous but segmented categorically. An example is a topographic map, used in earth science disciplines as well as by hikers, emergency rescue operations, and other endeavors requiring knowledge of terrain. Successful comprehension requires understanding that continuous elevation information is categorically encoded using contour lines, as well as skill in visualizing the three-dimensional shape of the terrain from the contour lines. In Experiment 1, we investigated whether novices would benefit from pointing and tracing gestures that focus attention on contour lines and/or from three-dimensional shape gestures used in conjunction with three-dimensional models. Pointing and tracing facilitated understanding relative to text-only instruction as well as no instruction comparison groups, but shape gestures only helped understanding relative to the no instruction comparison group. Directing attention to the contour lines may help both in code breaking (seeing how the lines encode elevation) and in shape inference (seeing how the overall configuration of lines encodes shape). In Experiment 2, we varied the language paired with pointing and tracing gestures; key phrases focused either on elevation information or on visualizing shape. Participants did better on items regarding elevation when language highlighted elevation and better on items requiring shape when language highlighted shape. Thus, focusing attention using pointing and tracing gestures on contour lines may establish the foundation on which language can build to support learning.

## Significance

A critical skill in many earth science disciplines is learning to interpret topographic maps that use contour lines to encode elevation information and represent the three dimensional shape of structures in the real world. Novices struggle with this task. Here we manipulate two spatial learning tools, gesture and language, to facilitate topographic map comprehension. This study reveals principles for instruction on topographic map understanding that could be adapted to other layered maps (e.g., weather maps).

## Background

**Fig. 1:**
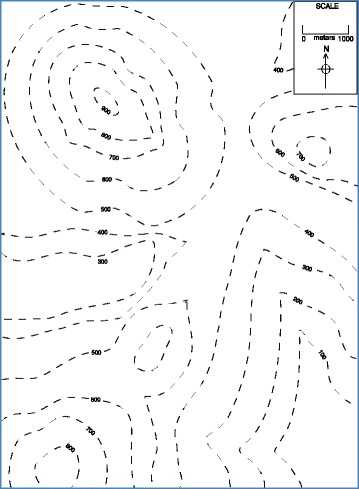
*Sample topographic map.* An image of a sample topographic map used during instruction. It is a topographic map adapted from Bennison and Moseley (2003) that contains contour patterns representing a hill, a valley, and steep and shallow slopes

Novices struggle to interpret maps that show information about continuous dimensions (typically latitude and longitude), layered with information that is inherently continuous but segmented categorically. An example is a topographic map, which is used in earth science disciplines as well as by hikers, emergency rescue operations, and other endeavors requiring knowledge of terrain. An example of a topographic map is provided in Fig. 1. Successful comprehension requires understanding that continuous elevation information is categorically encoded using contour lines, as well as skill in visualizing the three-dimensional shape of the terrain from the contour lines. Topographic maps are a “graphic representation of the three dimensional configuration of the earth” (Geographic Information Technology Training Alliance, 2016) and are commonly used to help gain a three-dimensional understanding of the landscape of a region (Dennis, 1972). Although frequently used by experts, these maps are particularly difficult for students to comprehend (e.g., Clark *et al*., 2008; Rapp, Culpepper, Kirkby, & Morin, 2007).

Two aspects of the correspondence between topographic maps and the world are notable. First, the correspondence between elevation values and elevation in the world is hard to grasp. Elevation relative to sea level is a challenging concept in the absence of a visible sea. Second, the correspondence between patterns of contour lines on the map and surfaces in the world is difficult to grasp because there are no visible feature boundaries in the world that align to single contours on a topographic map (e.g., a line that shows where a hill starts or stops). Rather, the shape of three-dimensional structures can be inferred from the relative shape and location of the contour lines (e.g., closely spaced contours represent steep slopes, and widely spaced contours represent shallow slopes).

Topographic maps are members of the class of diagrams called *isograms* (Brooks, 1916). Another example is a weather map where the continuous dimension of temperature is categorically displayed as regions where the temperature falls within specific values, and contour lines are drawn at the boundaries of those regions so that a contour connects points that have equivalent temperature values (Talman, 1910). In this case, the form of the iso-temperature contours preserves aspects of the spatial structure of the temperature distribution over the region, just as elevation contours preserve aspects of the spatial structure of the terrain. Yet another example is weather maps showing high and low pressure systems with contours to categorically indicate specific barometric pressure values. In general, all types of isograms, which encode one dimension categorically by segmenting continuous values arbitrarily, and visually preserve and continuously present other properties, are complex and can be difficult to learn and use (Hegarty, Canham, & Fabrikant, 2010; Taylor, Renshaw, & Choi, 2004).

Much prior research on learning to use topographic maps has focused on trying to facilitate comprehension of the topography by providing the user with additional visual information on the map, such as with shaded relief (e.g., Phillips, Lucia, & Skelton, 1975; Potash, Farrell, & Jeffrey, 1978; Rapp *et al*., 2007). A critical finding from this work is that the benefits of additional visual information are limited to reasoning about specific kinds of topographic map problems (e.g., shaded relief shows a moderate improvement over contour lines for landscape visualization tasks, but decreases accuracy for spot elevation problems; Potash *et al*., 1978). Furthermore, although visual enhancements have been found to help students understand other kinds of contour diagrams (Taylor *et al*., 2004), the effects of additional visual information during learning on students’ skills for topographic map use later on is still unknown.

Other attempts to support learning to interpret topographic maps have tried various instructional strategies to make salient the line of equal elevation on a three-dimensional model (e.g., students submerge a model in water and record where the water meets the model; Piburn *et al*., 2002; Trasper, 2010). We know of no experimental test on the efficacy of this approach. However, given the difficulty some students experience with visualizing water level (Liben, Christensen, & Kastens, 2010), students may well have difficulty applying the lesson to visualize an imaginary waterline on topography in the field.

The aim of the present work was to further our understanding of how people learn about shape and elevation information represented on a topographic map. Specifically, we explored how students learn that elevation information is encoded using contour lines, and how they learn that the two-dimensional patterns created using groups of contour lines on a topographic map depict the shapes of three-dimensional structures in the real world. We took advantage of two tools commonly used in science, technology, engineering, and mathematics (STEM) education to support students learning complex spatial tasks: gestures, which are used to highlight and/or portray multifaceted spatial information; and language, which uses individual words to stand for categorical concepts (Atit *et al*., 2013; Jackendoff & Landau, 1991).

Gestures are movements of the body, usually the hands, which are produced when engaging in effortful cognitive activity, such as speaking or problem solving (Alibali, 2005). Common spatial activities, such as giving directions, often include gestures (e.g., Lavergne & Kimura, 1987), and domains of science that require communicating complex spatial ideas, such as geology, often make use of gestures (Liben *et al*., 2010). One common function of gestures is that they are used to focus attention to spatial information (e.g., Atit, Shipley, & Tikoff, 2014; Lozano & Tversky, 2006; Roth 2000). For example, pointing and tracing gestures can be used to draw the listener’s attention to critical pieces of information within the conversational space (e.g., Heiser, Tversky, & Silverman, 2004; Lozano & Tversky, 2006). Geoscience experts, when asked to interpret and explain complex diagrams, such as a geologic map, use pointing and tracing gestures to focus their students’ attention to important pieces of information on the map (Atit *et al*., 2013). Another common function of gestures is that they are used to convey complex three-dimensional spatial relationships (e.g., Alles & Riggs, 2011; Atit, Gagnier, & Shipley, 2015). Three-dimensional gestures are well suited to portray continuous complex three-dimensional spatial relations because they can depict information about the space, shape, form, and position of an object simultaneously (Atit *et al*., 2014). Instructors use three-dimensional gestures when describing three-dimensional forms represented on two-dimensional diagrams (Atit *et al*., 2013), and researchers have found that gesturing about three-dimensional spatial relationships bolsters students’ skill in reasoning about diagrams of three-dimensional spatial relations (Atit *et al*., 2015). These observations suggest that three-dimensional gestures could be used to help a student understand shape information on topographic maps by connecting the spatial relations within and between contour lines to the spatial relations of the structures in the world.

Language is also a useful tool for learning complex spatial relationships (e.g., Loewenstein & Gentner, 2005). Similar to the way elevation information is categorically encoded using contour lines on a topographic map, spatial information is categorically encoded and communicated using language (Jackendoff & Landau, 1991). For example, the word *cone* represents a category of three-dimensional geometric shapes that tapers smoothly from a point to a planar base. Thus, understanding the information transmitted through language requires the listener to form corresponding conceptual representations (e.g., Gentner, Özyürek, Gürcanli, & Goldin-Meadow, 2013).

To assess students’ understanding of how elevation is encoded on a topographic map, and how the three-dimensional shape of a terrain is depicted on the map, we employed two types of topographic map test items: those that emphasize understanding of how elevation is denoted using contour lines (i.e., elevation items), and those that emphasize comprehending the three-dimensional shape of the represented terrain (i.e., shape items). Previous work suggested a dissociation between items that required an understanding of elevation information and items that required an understanding of shape information; participants who used the word “elevation” in their written descriptions of practice maps did better in an assessment of topographic map comprehension (Newcombe *et al*., 2015).

In Experiment 1, we compared two interventions using two kinds of gestures routinely used by experts when explaining complex diagrams (Atit *et al*., 2013). One intervention used pointing and tracing gestures to focus students’ attention on contour lines representing elevation information on a topographic map. The second intervention used three-dimensional gestures and models to help students align the contour patterns on the map to the three-dimensional shape of the structure in the real world. In Experiment 2, we investigated the role of verbally providing conceptual frameworks that emphasized either elevation or shape information paired with the pointing and tracing gesture found to work best in Experiment 1.

## Experiment 1

Accurately using a topographic map requires a variety of skills including an understanding of scale, orientation, and viewing angle, and extracting three-dimensional information from a two-dimensional representation (Liben & Titus, 2012). Two skills are fundamental for successful map usage: (1) knowledge of how elevation information about the terrain is encoded using contour lines (i.e., elevation information); and (2) an understanding of how the two-dimensional contour patterns on a topographic map align to three-dimensional structures in the real world (i.e., shape information). A survey of the relevant literature suggests that novices have difficulty with both of these tasks (e.g., Clark *et al*., 2008; Rapp *et al*., 2007). In this experiment, we use two kinds of gestures, pointing and tracing gestures and three-dimensional gestures, both helpful in understanding complex two-dimensional diagrams (e.g., Atit *et al*., 2013; Atit *et al*., 2015), to bolster novices’ skills. We hypothesized that understanding elevation and understanding shape are two distinct skills, and thus the two different types of gestures (pointing and tracing versus three-dimensional shape gestures) will differentially support understanding of the different types of topographic map concepts (elevation versus shape). Specifically, we predicted that using pointing and tracing gestures (Pointing and Tracing group) during instruction to highlight individual contour lines would promote learning of how elevation is encoded by contours on a topographic map; we also predicted that using 3D gestures to map the spatial relations between the contour patterns on the map and the three-dimensional structure in the real world represented here using three-dimensional models (3D Gestures and Models group), would enhance topographic map terrain understanding in novices. In addition to the two instructional groups, we included two comparison groups: one that received basic text-based instruction on topographic maps, and one that received no instructions.

If pointing and tracing gestures help novices understand how contour lines are used to denote elevation information, the Pointing and Tracing group should perform better than the other three groups on the elevation items on the measure of topographic map understanding. If three-dimensional gestures help novices understand how two-dimensional patterns on the map align to three-dimensional structures in the world, the 3D Gestures and Models group should perform better than the other three groups on the shape items in the same assessment. If gesturing in general facilitates topographic map understanding overall, both instructional groups should perform better than the comparison groups on the assessment.

Finally, to explore the relations between pre-existing skills and aptitude for learning how to use topographic maps, all participants completed three additional measures. One measure asked about the participants’ previous experience with topographic maps since experience has been found to influence how maps are processed by the user (Montello, Sullivan, & Pick, 1994). A second measure, the Water Level Test (WLT), assessed participants’ understanding of horizontality (e.g. Kastens & Liben, 2007; Liben & Golbeck, 1980). As contour lines on a topographic map represent horizontal planes of equal elevation, skill in understanding topographic maps could be related to skill in reasoning about horizontal planes. Lastly, since using a topographic map often involves coordinating the map user’s location in the world with a location on the map, skill in envisioning what would be seen from that perspective (Ishikawa & Kastens, 2005) might also be critical. Perspective-taking skill was assessed with the Spatial Orientation Test (SOT) developed by Hegarty and Waller (2004).

## Methods

### Participants

Newcombe *et al*. (2015) found that undergraduate men perform better than women on the Topographic Map Assessment (TMA) instrument. Similarly, Boardman (1989) found gender differences in topographic map comprehension in 11 to 14-year-old children. Thus, in this study we focus on novice women to avoid potential ceiling effects. We recruited 272 female undergraduate psychology students (mean age: 20.58 years, age range: 18 to 53 years) through our university’s Psychology Research Pool. Participants provided informed consent and received credit towards a research participation requirement for their involvement in the study.

### Materials

#### Map Experience Survey

The Map Experience Survey is a six-item survey adapted from Weisberg, Newcombe, and Shipley (2013) designed to assess a participant’s previous experience with topographic and planimetric maps (maps that represent only horizontal positions of surface features of a region; Gilhooly, Wood, Kinnear, & Green, 1988). It includes three *yes* or *no* questions such as “Do you like to hike or camp?” and three Likert-type items such as, “Rate your experience with maps in general” where responses ranged from 1 (“no experience”) to 7 (“a lot of experience”). The Map Experience Survey is provided in Appendix A.

#### Spatial Orientation Test

The SOT is a test designed to assess perspective-taking skill. A participant’s task is to imagine standing at the position of one object in a seven-object display (the station point) facing another object, and then to indicate the direction to a third object. The participant responds by drawing an arrow showing the corresponding direction on a blank circle where the station point is located in the center and the facing object is located at the top. Five minutes are provided to complete the 12-item test. The participant’s score on each item is the absolute deviation in degrees between her response and the correct direction to the target (Hegarty & Waller, 2004).

#### Water Level Test

The WLT assesses skill in identifying a stable horizontal axis in spite of a conflicting visual context (Piaget & Inhelder, 1956). A participant is presented with line drawings of six straight-sided bottles tipped from upright to the right or left. The task is to draw a line inside each bottle to show the location of the water if the bottle were half full and held in the position shown. Lines that deviate more than 5 degrees from horizontal are categorized as errors (Liben & Golbeck, 1980).

#### Introduction to Topographic Maps

“Introduction to Topographic Maps” is a two-page handout that provides background and instructions on how to interpret topographic maps. It was written based on simple, concise descriptions culled from online resources. Expert geoscientists who regularly teach how to use these maps approved the final version prior to its use in this study. It describes the different uses of topographic maps and introduces the concept that contour lines are used to represent elevation (Jacovina, Ormand, Shipley, & Weisberg, 2014).

#### Sample topographic map

The two-dimensional sample topographic map is a topographic map adapted from Bennison and Moseley (2003) that depicts three simple topographic forms (*hill, slope,* and *valley*), which are commonly taught in introductory geoscience classrooms (e.g. Bennison & Moseley, 2003; Busch, 2011). These forms were the focus of instruction for both the Pointing and Tracing group and the 3D Gestures and Models group. The map is shown in Fig. 1.

#### Three-dimensional stepped contour models

**Fig. 2:**
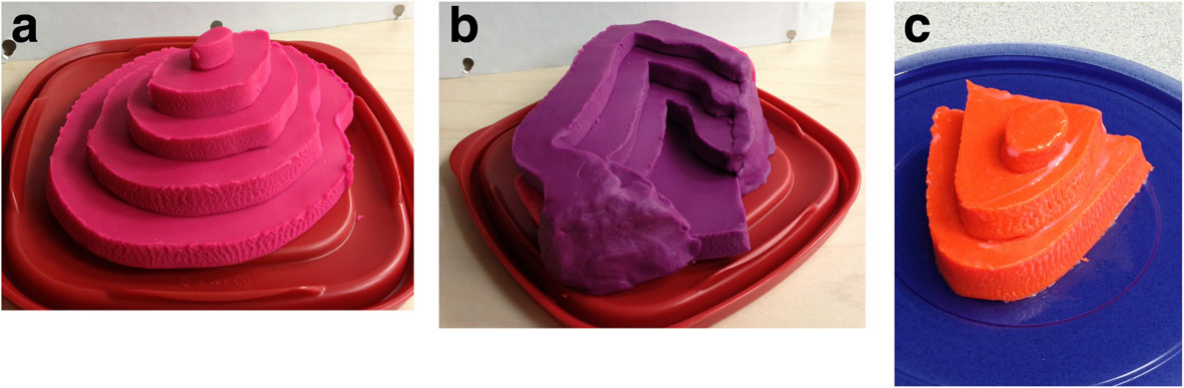
*Three-dimensional stepped contour models for Experiment 1.* Images of the three aligned models of the structures represented on the sample topographic map: **a** hill, **b** valley, and **c** steep and shallow slopes

Nine three-dimensional stepped contour models made from flat layers of Play-Doh (a soft modeling compound) were used in this study. Flat layers were used to visually emphasize that contour lines represent specific elevations. Pilot work with novice topographic map users showed that students struggled to align smooth models to the map, and so models with flat layers were used here. Four of the models were aligned with the sample topographic map, and the remaining five depicted additional examples of mountains, valleys, ridges, and slopes not portrayed on the map. Of the four aligned models, one model depicted the entire map, and the remaining three depicted specific structures: a hill, a valley, and a steep slope and a shallow slope. Images of three of the four aligned models (the model of a hill, the model of a valley, and the model of a steep slope and a shallow slope) are shown in Fig. 2. The five remaining models, those not aligned with the sample topographic map, were models of a hill, a valley, a ridge, a steep slope, and a shallow slope.

#### Practice problems

**Fig. 3:**
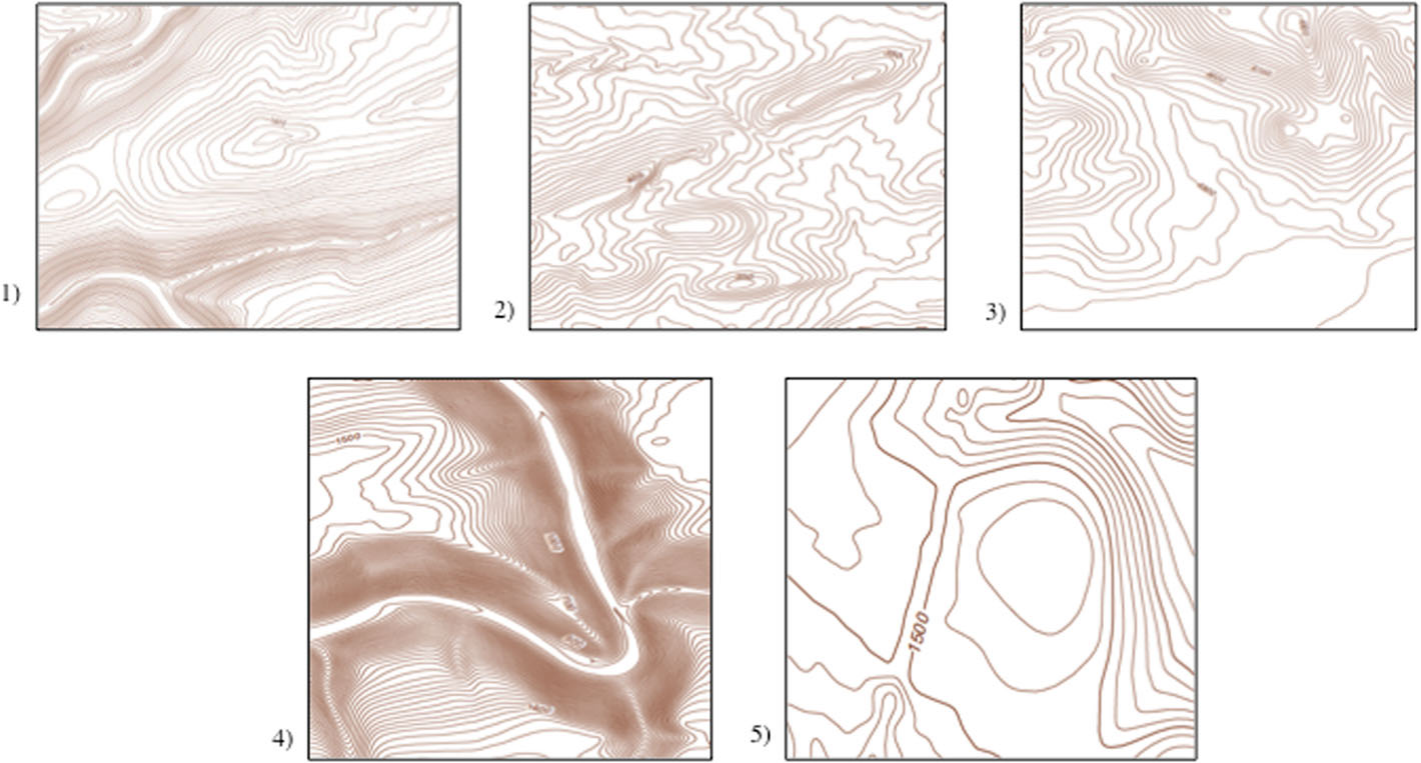
*Practice problem maps.* Five maps used for practice problems in Experiment 1

To provide the Pointing and Tracing group and the 3D Gestures and Models group practice interpreting topographic maps, practice problems were created from five US Geological Survey (USGS) maps (shown in Fig. 3). For each map, participants in the Pointing and Tracing group pointed or traced and verbally labeled each structure, and participants in the 3D Gestures and Models group made the three-dimensional gesture and identified the model of the structure.

#### Topographic Map Assessment

The TMA is an 18-problem paper assessment of topographic map use. Each problem presents a topographic map and requires either a discrete response (e.g., “Which elevation profile matches the cross-section of the line AB?”) or an open-ended response (e.g., “Imagine there is a stream that connects the circle and the square. Please draw the path you believe the stream would follow.”). No problems on the assessment ask participants to directly recall the structures learned during instruction. Five of the 18 problems include open-ended responses. The assessment is not timed. Six problems have more than one item (e.g., draw a path and provide an explanation), and for purposes of scoring these problems, each item was awarded one point (Newcombe *et al*., 2015).

### Scoring of the Topographic Map Assessment

The TMA is composed of a variety of problems that sample the different ways topographic maps are used by geoscientists when working in the field. To assess overall topographic map understanding, we summed the scores for all 18 problems on the assessment. Thus, the total number of points possible on the assessment was 28.

Five of the items in the measure asked participants to draw their responses. Three of the five open-ended items required drawing watercourses on a map, and the remaining two required drawing the route they would walk from one location to another. To ensure that these items were scored reliably, inter-rater reliability was established by having a second independent coder score a subset (20 %) of the responses for each item. Inter-rater agreement for all five items was high, all *k*’s >0.85 (*n* = 54 responses).

In addition, all of the items on the TMA were coded by two authors (SMW, KA) and two expert geoscientists, for whether each item was more likely to require knowledge about elevation information (i.e., elevation items) or knowledge about shape information (i.e., shape items). Agreement was high across the 28 items. The four raters reached consensus on 16 items (consensus coding), and three raters agreed on 22 items (majority coding). The remaining items were considered to substantively require both elevation and shape. To understand the effect of instruction type on item-type, items for which three or more raters agreed (majority coding) were used in the analyses (findings were substantively the same when analyses were restricted to the 16 consensus coding items). The three raters coded 13 items as elevation and nine items as shape. For ease of comparison, we conducted the item-type analyses on the proportion of items correct.

### Procedure

Participants were tested individually in a quiet room. After completing the consent process, all participants completed the Map Experience Survey, the WLT, and the SOT. After completing the measures, each person was assigned to one of four groups (68 participants in each group): the Pointing and Tracing group, the 3D Gestures and Models group, the Text-based Instruction group, and the No Instruction group. Those participants who responded with a “4” or higher to item 2 (“Rate your experience with topographic maps”) on the Map Experience Survey were classified as high topographic map experience and were evenly distributed across the four groups (*n*_3DGesturesandModels_ = 7, *n*_PointingandTracing_ = 7, *n*_Text-basedInstruction_ = 8, *n*_NoInstruction_ = 8).

After completing the Map Experience Survey, participants in the Pointing and Tracing, 3D Gestures and Models, and Text-based Instruction groups were asked to read the “Introduction to Topographic Maps” handout. Then, only the Pointing and Tracing group and the 3D Gestures and Models group received additional training. The Text-based Instruction group and the No Instruction groups received no additional instruction before completing the TMA.

The Pointing and Tracing group was presented with the sample topographic map. The experimenter pointed to the hill on the map and explained that, “the bull’s-eye pattern indicates that the topography in this region is shaped like a hill.” After pointing to the contour pattern, the experimenter traced each of the concentric closed contours within the structure on the map and told the participant to “imagine a hill to help visualize what the area looks like.” The experimenter then asked the participant to mirror her actions, making the participant highlight each contour line within the structure. After the participant traced each of the lines in the bull’s-eye pattern, the experimenter explained why this type of pattern represents a hill, “The bull’s-eye pattern on the map is equivalent to a hill shape (*again pointing to the bull’s-eye pattern*) because the elevation at the center of the bull’s-eye (*tracing the center closed contour line)* is higher than the elevation on the outer rings (*tracing the two outer closed contour lines)*, meaning that the center of the structure should be higher than the outside.” After instructions on the hill, participants received analogous instructions for a valley, and for steep and shallow slopes. During instruction about the valley, the experimenter noted that a similar contour pattern could represent a ridge but the direction of change in elevation would be reversed.

**Fig. 4:**
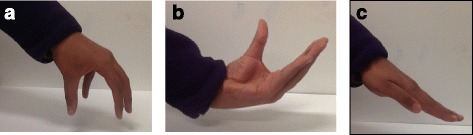
*Gestures for Experiment 1.* Images of the gestures used by the experimenter during instruction for the 3D Gestures and Models group: **a** the gesture of a hill, **b** the gesture of a valley, and **c** the gesture representing slope (the angle of the hand varied depending on whether a steep or shallow slope was represented)

The 3D Gestures and Models group was presented with the sample topographic map and the stepped contour model of the sample topographic map. After viewing the model, the experimenter focused the participant’s attention on individual structures. First, the experimenter pointed to the hill on the map and explained that the bull’s-eye pattern indicates that the topography in this region is shaped like a hill. After pointing to the contour pattern, the experimenter made a gesture of a hill (shown in Fig. 4a) and then presented the aligned model of the hill while stating “Whenever you see a bull’s-eye pattern like this one, you should shape your hand to represent a hill, and imagine a model of a hill, to help visualize what the area looks like.” She then used the gesture to spatially align the model to the contour pattern on the map. As she moved her hand from one representation to the other, she explained, “This gesture represents the structure of a hill like the one shown in this model (*the hill-shaped hand is placed over the model)*, and thus represents the structure shown on this map” (*the hill-shaped hand is placed over bull’s-eye pattern on the map*). The experimenter then asked the participant to mirror her actions to make the same *gesture*-to-*model*-to-*map* alignment. The experimenter further explained that the spatial relations of the shape of her hand are aligned with the two-dimensional pattern of the structure shown on the map, “The bull’s-eye pattern on the map is equivalent to this hill shape (*again makes a gesture of a hill*) because the elevation at the center of the bull’s-eye is higher than the elevation on the outer rings, meaning that the palm of your hand should be higher than your fingertips.” After learning about the hill, the participant in the 3D Gestures and Models group received analogous instructions for a valley, and for steep and shallow slopes. During instruction about the valley, the experimenter also noted that the same contour pattern could represent the structure of a ridge depending on the direction of change in elevation, and presented the participant with a gesture of a ridge.

After instructions about the hill, the valley, and the steep and shallow slopes were completed, participants in both the Pointing and Tracing group and 3D Gestures and Models group completed the set of five practice problems. While completing the problems, the 3D Gestures and Models group was presented with the additional models of a hill, a valley, a ridge, a steep slope, and a shallow slope, and was asked to use those models in their responses. Both groups received feedback after completing each problem.

Participants in all four groups then completed the TMA. Before starting the assessment, the Pointing and Tracing group was reminded to imagine the relevant structures, and the 3D Gestures and Models group was reminded to use the gestures and think about the models while completing the assessment. The experimenter was not present in the room during the test.

## Results

### The effect of instruction on TMA performance

**Fig. 5:**
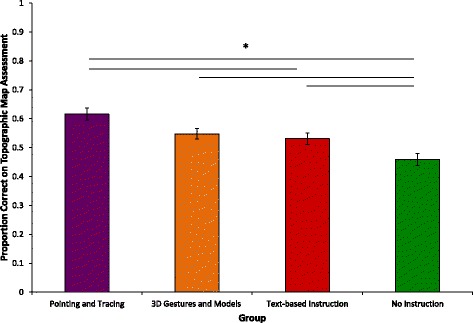
*Graph showing proportion correct on Topographic Map Assessment for each group in Experiment 1.* Bar graph showing proportion correct on the Topographic Map Assessment for the Pointing and Tracing group, 3D Gestures and Models group, Text-based Instruction group, and No Instruction group from Experiment 1. Cohen’s *d*’s for all pairwise comparisons are as follows: Pointing and Tracing versus 3D Gestures and Models (*d* = 0.44), Pointing and Tracing versus Text-based Instruction (*d* = 0.51), Pointing and Tracing versus No Instruction (*d* = 0.93), 3D Gestures and Models versus Text-based Instruction (*d* = 0.10), 3D Gestures and Models versus No Instruction (*d* = 0.56), Text-based Instruction versus No Instruction (*d* = 0.44). *p < .05

An analysis of variance (ANOVA) comparing participants’ responses to the item “Rate your experience with topographic maps,” on the Map Experience Survey in each condition verified that the groups did not differ in levels of topographic map experience, *F*(3, 268) = 0.29, *p* = .83, *ω* ^*2*^ = –0.008*.* Nor did they differ in performance on the SOT *F*(3, 268) = 0.54, *p* = .66, *ω* ^*2*^ = –0.005 or the WLT *F*(3, 268) = 0.17, *p* = .92, *ω* ^*2*^ = –0.009. Omega squared is an effect size statistic used when conducting an ANOVA (Tabachnick & Fidell, 2007). Thus, any difference in performance on the TMA was not a result of a difference in previous experience with topographic maps, perspective-taking skill, or an understanding of horizontality. Mean performance for each of the four groups is shown in Fig. 5.

To see if performance on the TMA (using the 22 items included in the majority coding) across the four groups varied by instruction and by item-type (elevation items and shape items), a mixed methods ANOVA was conducted with item-type as the within-subjects factor and instruction group as the between-subjects factor. The analysis revealed a reliable effect of group, *F*(3, 268) = 9.44, *p* < .001, partial *η* ^*2*^ = 0.10. Partial *η* ^*2*^ is an effect size statistic that is used when conducting an ANOVA with multiple independent variables, including a mixed methods ANOVA (Tabachnick & Fidell, 2007). Tukey post-hoc comparisons were used to identify differences in performance between the individual groups. The Pointing and Tracing (*M* = 0.62, *SD* = 0.17), the 3D Gestures and Models (*M* = 0.55, SD = 0.15), and the Text-based Instruction (*M* = 0.53, *SD* = 0.16) groups all performed significantly better than the No Instruction group (*M* = 0.46, *SD* = 0.17) on the TMA, all *p*’s < .05 (effect sizes for all significant pairwise comparisons are shown in Fig. 5). The Pointing and Tracing group also performed significantly better than the Text-based Instruction group (*p* < .05). There was no statistically significant difference in performance between the 3D Gestures and Models group and the Text-based Instruction group, or the 3D Gestures and Models group and the Pointing and Tracing group. Thus, instruction using pointing and tracing gestures to highlight the relationship between elevation and contour lines provided the most effective training, leading to a reliable boost in performance over text-based instruction alone when learning to use topographic maps.

**Fig. 6:**
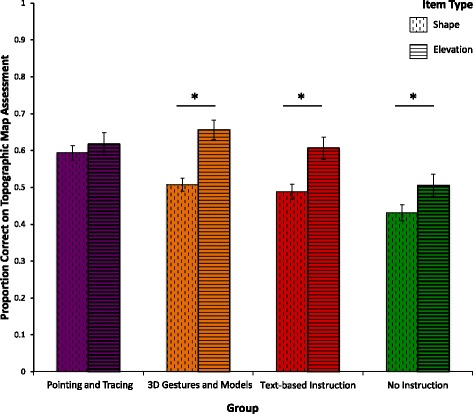
*Graph showing proportion correct on Topographic Map Assessment for groups by item-type for Experiment 1.* Bar graph showing proportion correct on the Topographic Map Assessment in the Pointing and Tracing group, 3D Gestures and Models group, Text-based Instruction group, and No Instruction group broken down by item-type (elevation versus shape items) from Experiment 1. Cohen’s *d*’s for all pairwise comparisons are as follows: elevation versus shape items for the Pointing and Tracing group (d=0.30), elevation versus shape items for the 3D Gestures and Models group (*d* = 0.57), and elevation versus shape items for the No Instruction group (*d* = 0.34). *p < .05

The analysis also revealed a significant effect of item-type. Overall, performance on the elevation items (*M* = 0.60, *SD* = 0.25) was significantly better than on the shape items (*M* = 0.51, *SD* = 0.25) on the TMA (as shown in Fig. 6): *F*(1, 268) = 43.13, *p* < .001*,* partial *η* ^*2*^ = 0.14. There was no significant interaction between condition and item-type: *F*(3, 268) = 0.95, *p* = .42*,* partial *η* ^*2*^ = 0.01.

We probed further to see whether our gesture interventions specifically affected performance on one type of item by focusing only on the gesturing conditions. In this analysis (again using majority coding), group (Pointing and Tracing and 3D Gestures and Models) was the between-subjects factor and item-type (elevation and shape) was the within-subjects factor. Results again indicated a significant effect of group (*F*(1, 134) = 4.64, *p* = .03, partial *η* ^*2*^ = 0.03), and a significant effect of item-type (*F*(1, 134) = 21.56, *p* < .001, partial *η* ^*2*^ = 0.14) but no interaction. Apparently, instruction focusing attention using pointing and tracing gestures to highlight contour lines improved participants’ performance on the TMA overall, on both elevation and shape items.

### Relations between individual difference measures and performance on the TMA

To explore the relationships among participants’ topographic map understanding and other pre-existing skills, correlations were calculated between performance on the TMA (using the 22 items included in the majority coding of the TMA) and previous map experience, the SOT, and the WLT (all correlations were interpreted using Cohen’s, 1988, conventions). A summary of the correlations, means, and standard deviations between each of the measures is shown in Table 1. Since the three Likert-type items on the Map Experience Survey were strongly correlated (all *r*’s >0.47, all *p*’s < .001), and Cronbach’s alpha was 0.78, the average of the three responses was used as a “previous map experience” composite score for all analyses. Performance on the TMA was weakly but significantly correlated with previous map experience (*r*(270) = 0.16, *p* = .01) and performance on the WLT (*r*(270) = 0.24, *p* < .001). Performance on the TMA was more robustly correlated with performance on the SOT (*r*(270) = –0.37, *p* < .001).

**Table 1:** Summary of correlations, means, and standard deviations for Experiment 1

Measure	1.Topographic Map Assessment	2. Map experience	3. Spatial Orientation Test (perspective taking)	4. Water Level Test	Mean	Standard deviation
1. TMA					0.54	0.17
2. Map experience	0.16**				2.60	1.17
3. SOT (perspective taking)	–0.37***	–0.21***			54.25	27.96
4. WLT	0.24***	0.10	–0.37***		0.37	0.30

Summary of correlations, means, and standard deviations on the Topographic Map Assessment, previous map experience, the Spatial Orientation Test, and the Water Level Test. *SOT* Spatial Orientation Test, *TMA* Topographic Map Assessment, *WLT* Water Level Test. Note. **p < .01, ***p < .001

## Discussion

Results from this study suggest that focusing attention on individual contour lines using pointing and tracing gestures facilitates topographic map understanding in novices. Women who received instruction using pointing and tracing gestures to emphasize that contour lines are used to represent elevation information on a topographic map performed better overall on a measure of topographic map use than women who received no instruction or basic written instructions. By contrast, the shape gestures paired with the models showed no difference in improvement from text-only instruction. In addition, there was no interaction of the type of instruction with the specific types of topographic map reading items.

We had hypothesized that pointing and tracing gestures, which are commonly used to focus attention to local spatial information (e.g., Atit *et al*., 2013; Lozano & Tversky, 2006), would bolster novices’ understanding of elevation information, while three-dimensional gestures, which are commonly used to convey complex three-dimensional continuous spatial relations (e.g., Atit *et al*., 2013; Atit *et al*., 2014), would bolster novices’ understanding of shape information. However, the results from the experiment suggest that using pointing and tracing gestures to focus attention on contour lines facilitated understanding for both types of information. Perhaps this overall boost occurred because untrained novices have trouble identifying any meaningful information in contour lines (e.g., Chang, Antes, & Lenzen, 1985). By highlighting individual contour lines, which encode categories of elevation values, within meaningful patterns of contour lines, which encode terrain shape, the novices may have been better able to make sense and extract useful information about both elevation and shape from the topographic maps, and to ignore the irrelevant information. That is, drawing attention to a contour line may not only highlight it, but also segment groups of contour lines, thus affecting both elevation and shape understanding.

Gestures are, of course, not the only way that people receive instruction on map comprehension. They also read and listen to verbal instruction. Acquiring a linguistic understanding of a concept may provide students with a representational system that can help them grasp complex and novel information (e.g., Gentner *et al*., 2013). Gestures and language together create a single integrated system of meaning expression where each can add or constrain the spatial meaning of the other (McNeill, 1992). In Experiment 1, the speech during instruction contained information about both elevation (that contour lines are used to encode elevation information), and shape (that the patterns of contour lines resemble the shape of the three-dimensional structure in the real world). In Experiment 2, we separated these two concepts to determine whether the concepts conveyed in the speech accompanying pointing and tracing gestures (the most effective gestures in Experiment 1) would differentially facilitate participants’ performance on the two specific kinds of topographic map items (elevation and shape items).

## Experiment 2

In Experiment 2, we explore how the concepts conveyed in speech influence the information processed by novices when learning to understand topographic maps; we do this by varying the narrative accompanying pointing and tracing gestures to emphasize elevation information (Elevation Language group) or shape information (Shape Language group). If an understanding of elevation and an understanding of shape are distinct, and if they can be influenced by language, topographic map comprehension should be differentially improved on items that relied on the emphasized concept. On the other hand, any type of conceptual focus may provide learners an opportunity to associate contour lines with the concept of elevation and to derive three-dimensional shape. If this prediction is correct, participants in both language conditions should show improvements across both elevation and shape items.

## Methods

### Participants

We recruited 58 participants from an undergraduate psychology department at a research university to complete a 1-hour study in exchange for class credit. Due to the large effect size in Experiment 1 between the Pointing and Tracing group and the Text-based Instruction group (*d* = 0.95), we aimed for a sample size of 19 participants per condition (with 80 % power and an alpha = .05, using G*Power 3.1.9.2; Faul, Erdfelder, Buchner, & Lang, 2009) but had to drop four participants due to experimenter error. Just as in Experiment 1, only female participants were recruited for this study. Participants were between the ages of 18 and 32 years (*M* = 20.76, *SD* = 2.90).

### Materials

The following materials were the same as those used in Experiment 1: Map Experience Survey, TMA, sample topographic map, and practice problems. The “Introduction to Topographic Maps” handout was omitted to allow comparison between the current study and the No Instruction condition from Experiment 1. The interventions were adapted or created as described below.

#### Materials developed for Experiment 2

**Fig. 7:**
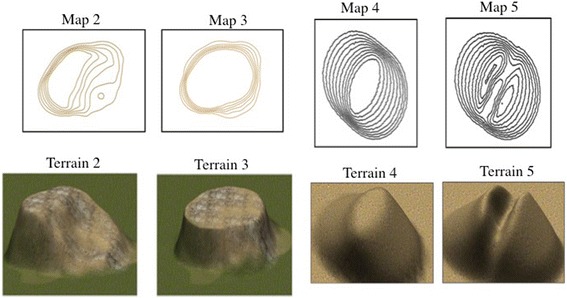
*Maps from Experiment 2.* Two sets of maps used during instruction in Experiment 2. Participants were instructed to think about how the topographic maps represented elevation in the three-dimensional terrain (Elevation Language group), how the two-dimensional shapes of the contour lines represented the three-dimensional shape of the terrain (Shape Language group), or explain how the maps and terrains related to one another (Open-ended group). The topographic maps are shown in the *top row* and the corresponding terrain image is shown *below*. Verified by geoscience experts, these terrains can be described as follows: (2) collapsed mesa, (3) mesa, (4) hill, (5) divided hill. Scripts used by the experimenter that accompany these maps are provided in Appendix B

We developed materials for Experiment 2 by adapting those from Experiment 1 and a pilot study (Newcombe *et al*., 2015), all of which were the same across all three conditions for Experiment 2 (except for what was said by the experimenters). The sample topographic map from Experiment 1 was used to introduce topographic maps. Next, two sets of topographic maps and terrain visualizations derived from virtual environments were used to illustrate the maps in more detail (shown in Fig. 7). Each set consisted of two maps and terrain visualizations, which were identical except for one change (i.e., two landscapes that are identical except that one features a hill, whereas the other features a divided hill with a valley down the center). Finally, the practice problems from Experiment 1 were shown to participants. While the materials were similar to those in Experiment 1, we changed the speech accompanying the gestures as described below.

#### Interventions

We devised three conditions, two of which conveyed different concepts in speech, in a between-subject design. Participants in the Elevation Language group were told that each contour line represents one value of elevation, and to consider that the concept of elevation implies that as contour lines are closer together, elevation changes more quickly. Participants in the Shape Language group were encouraged to focus on the shape of the contour lines, and imagine how they might look in three dimensions. In both conditions, participants imitated the gestures made by the experimenter, and then answered questions about a set of practice maps using their own gestures. Participants in a third, no instruction condition received no instructions on how to interpret the maps, but saw the same stimuli, and were asked open-ended questions about each map. Gesturing by the participant was neither encouraged nor discouraged. Condition assignment was pseudo-randomized and counter-balanced for map experience based on reports of experience with topographic maps (participants’ response to “Rate your experience with topographic maps” on the Map Experience Survey).

The language interventions consisted of two parts: an experimenter-led script, followed by open-ended questions. For the experimenter-led script: the experimenter guided participants of the Elevation Language group through a series of sample topographic maps, describing how the lines provided information on how to analyze the maps and determine the elevation of specific contour lines; the experimenter guided participants of the Shape Language group through a the same sample topographic maps, describing how the lines provided information on how to analyze the maps and how the shape of the lines would allow them to visualize the three-dimensional shape of the terrain surface. The experimenter-led script for both the Elevation Language group and the Shape Language group is provided in Appendix B.

The experimenter read from a script while the participant looked at the maps being described. Throughout the reading of the script, the experimenter gestured on the maps by pointing out various features and tracing the contour lines. For the experimenter-led portion of the intervention, the maps and gestures were identical for the Elevation Language group and the Shape Language group. The script itself was created by adapting the script used with the Pointing and Tracing group in Experiment 1 to emphasize elevation information. An analogous script for shape was then generated by revising each sentence of the script to emphasize shape information, specifically emphasizing transitioning from a two-dimensional pattern to a three-dimensional shape. The two resulting scripts were then matched in structure as closely possible. Both scripts include definitions, examples, and comparisons of either elevation or shape. The Elevation Language group’s script mentions the word elevation 31 times, whereas the Shape Language group’s script mentions the word shape 32 times.

The second part of the intervention, the practice problems, was also adapted from Experiment 1. The questions were designed to focus the participant’s attention on how the contour lines provided information about that condition’s concept. The Elevation Language group’s questions focused on two elevation-related concepts: (a) that each line depicts one level of elevation, and (b) that elevation changes as you move across and between contour lines. The Shape Language group’s questions focused on two three-dimensional shape-related concepts: (a) that the shapes of the lines in two dimensions could be used to predict the shape of the surface in three dimensions, and (b) that the three-dimensional shape of the surface changes as a function of the change in the shape of the lines. The Elevation Language group’s questions mention the word elevation seven times, while the Shape Language group’s questions mention the word shape six times.

The Open-ended group saw the same topographic maps as the two speech groups, and the participants were simply asked to describe what they saw on the maps with requests such as “Take a look at this topographic map, and describe in as much detail as possible what you think the terrain it represents looks like.” When presented with maps 2 and 3 and terrains 2 and 3 (shown in Fig. 7), the participants were asked to compare and contrast both maps and the terrains represented by the maps. When participants were presented with maps 4 and 5 and terrains 4 and 5 (shown in Fig. 7), again, they were asked to compare and contrast the maps and the terrains they represented. The experimenter prompted participants by saying, “Is there anything else you notice about the map(s)?” The complete script for the experimenter-led portion of the experiment is provided in Appendix B. After completing these tasks, participants in the Open-ended group also completed open-ended questions using the same practice maps as those presented to the intervention groups. For the open-ended questions, instead of focusing on a particular type of information, participants were asked to describe each topographic map and were only prompted to give more detail.

### Procedure

Participants were tested individually in a quiet room. After completing the consent process, all participants completed the Map Experience Survey. After completing the measure, each participant was assigned to one of the three conditions pseudo-randomly accounting for high or low experience with topographic maps. This resulted in 18 participants in the Elevation Language group, 19 participants in the Shape Language group, and 17 participants in the Open-ended group. High experience with topographic maps was rare and was evenly distributed across the three groups as in Experiment 1 (*n*_ElevationLanguage_ = 2, *n*_ShapeLanguage_ = 3, *n*_Open-ended_ = 2).

Participants in all three groups then completed the TMA. Before starting the assessment, each participant was reminded to focus on the concept emphasized during the intervention (elevation or shape). The experimenter was not present in the room during the test.

### Scoring of the TMA

Scoring of the TMA proceeded in the same manner as in Experiment 1, except where otherwise noted.

## Results

Using the subset of items on the TMA that were coded as either elevation items or shape items by three out of the four raters (majority coding), overall the Shape Language group (*M* = .59, *SD* = .20) and Elevation Language group (*M* = .61, *SD* = .12) numerically, but not significantly, outperformed the Open-ended group (*M* = .50, *SD* = .18), *F*(2, 51) = 2.10, *p* = .13, *ω* ^*2*^ = .04. The three groups were significantly different when all items from the TMA were included, with the Shape Language group (*M* = .64, *SD* = .15) and Elevation Language group (*M* = .66, *SD* = .11) significantly outperforming the Open-ended group (*M* = .54, *SD* = .16), *F*(2,51) = 3.28, *p* = .04, *ω* ^*2*^ = .08.

**Fig. 8:**
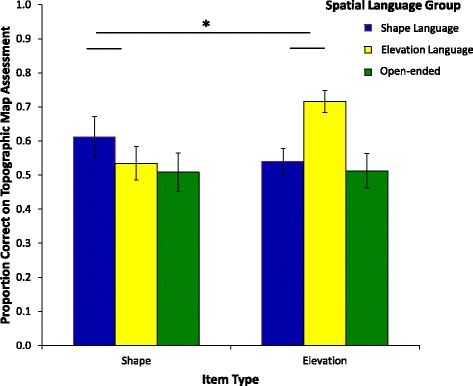
*Graph showing proportion correct on Topographic Map Assessment for groups by item-type for Experiment 2.* Bar graph showing proportion correct on the Topographic Map Assessment in the Elevation Language group, Shape Language group, and Open-ended group broken down by item-type (elevation versus shape items) from Experiment 2. Significance bars indicate a significant crossover interaction between item-type (elevation versus shape) and spatial language condition (Elevation Language versus Shape Language). *p < .05

To assess how the language intervention influenced spatial learning, we ran a two-factor ANOVA with group (Elevation Language group and Shape Language group) as a between-subjects factor and item-type (elevation and shape) as a within-subjects factor for the 22 TMA items where there was majority agreement on coding (shown in Fig. 8). We found a significant crossover interaction between item-type and speech condition, *F*(1, 35) = 10.87, *p* = .002, *η* ^*2*^ = 0.23. Eta squared is a measure of effect size used when conducting an ANOVA (Tabachnick & Fidell, 2007). Post-hoc within-group pair-wise comparisons indicated that the Shape Language group performed numerically but not significantly better on the shape items (*M* = .62, *SD* = .18) than on the elevation items (*M* = .53, *SD* = .29) *t*(18) = 1.65, *p* = .12, *d* = 0.36, while the Elevation Language group performed significantly better on the elevation items (*M* = .72, *SD* = .21) than on the shape items (*M* = .53, *SD* = .14) *t*(17) = 3.08, *p* = .007, *d* = 1.03. The Open-ended group did equally poorly on the shape items (*M* = .50, *SD* = .21) and the elevation items (*M* = .49, *SD* = .24) *t*(16) = 0.16, *p* = .88, *d* = 0.05 (all Cohen’s *d*’s, also a measure of effect size, were calculated using Lenhard & Lenhard’s, 2015, online effect size calculator). Finally, to determine how the Open-ended group compared to the Language groups on the untrained items, we compared the Shape Language group to the Open-ended group on elevation items, *t*(34), = 0.32, *p* = .75, *d* = 0.10, and the Elevation Language group to the Open-ended group on shape items, *t*(33) = 0.69, *p* = .50, *d* = 0.24.

To see if the two language intervention groups performed differently on each item-type, post-hoc pairwise comparisons were conducted. The analysis revealed that the Elevation Language group performed better than the Shape Language group on the elevation items (*t*(35) = 2.20, *p* = .035, *d* = 0.74), whereas the Shape Language group performed marginally better than the Elevation Language group on shape items (*t*(35) = 1.71, *p* = .096, *d* = 0.58). These differences in performance further suggest that improvement for each item-type varied depending on the verbal instructions received by the participants.

## Discussion

Results from this experiment suggest that the information conveyed in speech that accompanies gestures influences the type of information processed from topographic maps by novices. While it is possible that language alone would be sufficient to facilitate learning in novices, the accompanying pointing and tracing gestures seem likely to have guided attention toward the relevant contour lines, allowing language to specify how to interpret them. Subsequent studies could employ a comparison group hearing the instructions without any gestures to reveal if language alone shapes learning.

There are several ways in which this effect of language could occur. Language may provide a conceptual framework used to interpret the map (e.g., Gentner *et al*., 2013), it may affect learning strategies, and/or it may align spatial information between the map and the topographical structure represented (e.g., Loewenstein & Gentner, 2005). However, in considering the effects of language, it is important to note that there was little effect on the non-trained items (e.g., the Elevation Language group performed no worse on the shape items than the Open-ended group). That is, specific instructional language does not focus attention on one type of information to the detriment of processing the other type, nor does it seem to enhance general map comprehension.

Thus, facilitating topographic map comprehension probably requires explicitly communicating both elevation information and shape information, as we did in Experiment 1.

## General discussion

Our results add to our understanding of the complex role of gestures in processing spatial information (Atit *et al*., 2014; Heiser *et al*., 2004; Lozano & Tversky, 2006), the integration of gesture and spatial language (e.g., Goldin-Meadow, Kim, & Singer, 1999; Singer & Goldin-Meadow, 2005), and teaching novices to use topographic maps. Taken together, Experiments 1 and 2 suggest that information conveyed in speech and information conveyed in gestures interact in complex ways to influence students’ understanding of the encoding of elevation information and the shape of contour patterns.

Experiment 1 showed that not all kinds of gestures used by experts are helpful for novices. The pointing and tracing gestures that proved helpful may have worked similarly to basic code-cracking skills. Just as novice readers learn to associate sounds with visual symbols when the pairing between them is highlighted, in our study, using pointing and tracing gestures to highlight contour lines helped novice map users to associate contour lines with the elevation information they encode. On the other hand, novices in the 3D Gestures and Models group had difficulty making an association when three-dimensional gestures were used to highlight contour lines on a map. Why the three-dimensional gestures were less effective remains an open, but important, question because expert geologists commonly use three-dimensional gestures to highlight information associated with three-dimensional structures on geologic maps (Atit *et al*., 2013). It is possible that a three-dimensional gesture provides too much information, both form and location information, for novices to process and retain, while pointing and tracing gestures, which provided information only about contour lines, presented less information and may have been easier to process. In other words, the alignment between a contour line and a value of elevation is highlighted in a point and trace gesture, whereas a three-dimensional gesture conveys multiple mappings simultaneously.

An alternative and potentially interesting explanation is suggested by the finding of Experiment 2, that pointing and tracing gestures can support learning about three-dimensional shape when combined with a linguistic emphasis on shape. While the three-dimensional gestures and models were intended to encode three-dimensional spatial relations spatially, the gesture representation may have conveyed information that was too specific. For example, students may have interpreted the information conveyed literally rather than symbolically (e.g., as specific three-dimensional hand shape), even though the shape presented in the gesture did not perfectly align to the shape represented by the contour pattern. In contrast, pointing to the topographical map pattern and emphasizing to novices the shape of the lines in language may have allowed understanding because the abstract spatial relations encoded in language may have provided novices with a strategy to interpret the contour lines spatially. Understanding the interplay between gesture and language will be important for supporting learning in the classroom especially because field experts use both pointing and three-dimensional gestures in addition to speech when teaching complex spatial concepts.

Overall, Experiment 2 showed that specific verbal instructions, at least when paired with helpful gestures, facilitated specific skills: interpreting the meaning of contour lines in terms of elevation, or thinking about the shape of the represented terrain. Goldin-Meadow and colleagues have noted that a true understanding of the processing of information conveyed through both speech and gesture requires an understanding of the integration of both modalities (e.g., Goldin-Meadow *et al*., 1999; Singer & Goldin-Meadow, 2005). Here, we have shown that pointing and tracing gestures effectively highlight relevant and meaningful symbolic and spatial information, and that language can provide a framework for the kind of information that is learned. This finding suggests that, early in learning, gestures that guide attention to complex spatial information combined with conceptually focused speech are more helpful than gestures that refine spatial concepts.

In these experiments, we did not examine participants’ spontaneous usage of either modality. Previous research has found that participants use more spatial content in their speech when they are allowed to gesture (Rauscher, Krauss, & Chen, 1996), and that the timing of a student’s gestures relative to his or her speech is indicative of where he or she is in the learning process: gestures tend to precede speech early in the learning of a concept (Crowder, 1996). Thus, future studies should consider how a novice’s spontaneous speech and gestures may influence their thinking and provide insight into how spatial concepts are formed.

In addition to topographic maps, there are a variety of diagrams that employ contour lines to represent continuous information both continuously and discontinuously (e.g., isotherm maps). An important future direction for research would be to examine how students learn to understand different kinds of isograms, and how experience with the diagrams in the form of speech and gestures influences learning. Beyond topographic maps specifically, and isograms more generally, conceptually focused speech and highlighting gestures might be useful to teach disciplinary diagrams across the STEM disciplines. As contour lines are employed to represent a wide range of content, such as three-dimensional mathematical functions and chemical state-change boundaries, it is critical to understand how these educational tools can be applied to potentially increase the effectiveness and efficiency of diagram education.

## Conclusions

We found evidence that when learning to interpret diagrams representing continuous information both continuously and discontinuously, the use of gestures and speech to emphasize specific aspects of the diagram is critical for the listener’s understanding. Pointing and tracing gestures can be used to focus the listener’s attention to relevant elevation information denoted by contour lines on a topographic map. Furthermore, focused conceptual information in the accompanying speech can help the learner understand how to use the pertinent information. Extant research on topographic map learning has largely tried to boost students’ understanding for these diagrams by providing additional visual information on the map. Here, instead of altering the diagram, we employ two tools that are regularly used in everyday conversation and while solving complex spatial problems, speech and gestures, to help students understand topographic maps. As diagram interpretation is a critical skill in many STEM disciplines, understanding how these tools can be effectively used to teach them may have broader implications for learning in STEM classrooms.

## Appendix A

### Map Experience Survey

1. Do you like to hike or camp (yes/no)?
2. Rate your experience with topographic maps:
  No experience 1 2 3 4 5 6 7 A lot of experience
3. Do you have any experience with, or have you ever taken a course on orienteering?
4. Have you ever taken a geology, geography, or geosciences course? If so, please provide the course name and whether topographic maps were taught or used.
5. Rate your experience with maps in general:
  No experience 1 2 3 4 5 6 7 A lot of experience
6. Rate how much you enjoy looking at, reading, or using maps in general:
  No enjoyment 1 2 3 4 5 6 7 A lot of enjoyment

## Appendix B

### Experimenter-Led Instructional Script for the Elevation Language Group

**(REFERRING TO THE SAMPLE TOPOGRAPHIC MAP)** Okay, let’s focus on the top left corner of the map **(POINT TO BULL’S-EYE PATTERN ON MAP).** Each contour line is one value of elevation. The number tells you how high above sea level the area *right on the line* is. **(POINT-AND-TRACE THIRD LINE)**. Each line represents a different elevation, so you can tell that the ground is getting higher **(POINT-AND-TRACE ONE LINE TOWARD MIDDLE)**, or lower in elevation **(POINT-AND-TRACE LINE TOWARD OUTSIDE)**. In between the lines, the elevation of the ground changes, like from here **(POINT-AND-TRACE FROM ONE LINE)** to here (**ACROSS WHITE SPACE TO ANOTHER LINE)**.

Now, let’s focus on the right side of the map **(POINT TO** ***STEEP*** **AND** ***SHALLOW*** **SLOPE REGION ON MAP).**

In this region, you have an area where the lines are farther apart next to an area where the lines are closer together. When the lines are closer together, the elevation is changing quickly, meaning the region has a *steep* slope **(TRACE PERPENDICULAR ACROSS THE LINES)**. When the lines are farther apart, the elevation is changing slowly, meaning the region has a *shallow* slope **(TRACE PERPENDICULAR ACROSS THE LINES)**.

**(REFERRING TO MAPS 2 AND 3)** Now, we’re going to compare two examples to each other, and get a chance to see how the elevation looks in a simulated terrain. Take a moment and compare the maps, above, with their terrains, below. Note that the two maps are the same, except for how the elevation changes.

Notice how the map on the right has contour lines that are very close together **(POINT AND TRACE CONTOUR LINES).** The elevation is changing rapidly on this part of the map. Now look at the map on the left. (**POINT AND TRACE CONTOUR LINES)**. The elevation changes slowly as you move along, because compared to these lines (**POINT BACK AT MAP ON RIGHT)**, the lines are farther apart.

**(REFERRING TO MAPS 4 AND 5)** Here is another example. Again, we’re going to compare two examples to each other, and get a chance to see how the elevation looks in a simulated terrain. Take a moment and compare the maps, above, with their terrains, below. Note that the two maps are the same, except for how the elevation changes.

Start at the top left of the map on the left, notice how the elevation increases from left to right **(POINT AND TRACE EACH CONTOUR LINE ON LEFT SIDE)**, then, inside the center contour line, the elevation stays the same **(POINT AND TRACE INNERMOST CONTOUR),** then decreases **(POINT AND TRACE EACH CONTOUR LINE ON RIGHT SIDE).** The map on the right is somewhat different. This time, the elevation is the same at different parts of each little peak **(POINT AND TRACE EACH LITTLE PEAK)**.

### Experimenter-Led Instructional Script for the Shape Language Group

**(REFERRING TO THE SAMPLE TOPOGRAPHIC MAP)** Okay, let’s focus on the top left corner of the map **(POINT TO BULL’S-EYE PATTERN ON MAP).** Each contour line connects with itself to make a two-dimensional shape. The shape of the lines tells you about the 3D shape of the terrain. **(POINT-AND-TRACE THIRD LINE)**. Each line represents a different area of the ground, so you can tell what shape that part of the terrain is **(POINT-AND-TRACE ONE LINE TOWARD MIDDLE)**, compared to other parts of the terrain **(POINT-AND-TRACE LINE TOWARD OUTSIDE)**. Now connect the lines and visualize how the shape of the terrain changes, like from here **(POINT-AND-TRACE FROM ONE LINE)** to here (**ACROSS WHITE SPACE TO ANOTHER LINE)**.

Now, let’s focus on the right side of the map **(POINT TO** ***STEEP*** **AND** ***SHALLOW*** **SLOPE REGION ON MAP).**

In this region, you have an area where the lines are farther apart next to an area where the lines are closer together. When the lines are closer together, the 3D shape of the terrain is steep, and changes quickly **(TRACE PERPENDICULAR ACROSS THE LINES)**. When the lines are farther apart, the shape of the terrain is shallow, and changes slowly **(TRACE PERPENDICULAR ACROSS THE LINES)**.

**(REFERRING TO MAPS 2 AND 3)** Now, we’re going to compare two examples to each other, and get a chance to see how the shapes look in a simulated terrain. Take a moment and compare the maps, above, with their terrains, below. Note that the two maps are the same, except for how the shape changes.

Notice how the outermost line is an oval **(POINT AND TRACE CONTOUR LINES).** The shape of the bottom part of the terrain is also an oval. Now, notice the shape of the contour line at the top. It looks like a half circle. The top of the terrain also looks like a half circle. The shape of the terrain in the image matches the shape of the terrain on the contour map. Now look at the map on the right. (**POINT AND TRACE CONTOUR LINES)**. The shape of the terrain, again, matches the shape of the map, just like the pair on the left (**POINT AT MAP ON RIGHT)**.

(**REFERRING TO MAPS 4 AND 5)** Here is another example. Again, we’re going to compare two examples to each other, and get a chance to see how the shapes look in a simulated terrain. Take a moment and compare the maps, above, with their terrains, below. Note that the two maps are the same, except for how the shape changes.

Notice how the shape of the map on the right matches the shape of the terrain below it. The left side is rounded **(POINT AND TRACE EACH CONTOUR LINE ON LEFT SIDE)**, the top is an oval **(POINT AND TRACE INNERMOST CONTOUR)**, then the right side is rounded again **(POINT AND TRACE EACH CONTOUR LINE ON RIGHT SIDE).** The map on the right is somewhat different. This time, the shape of the contour lines forms two smaller ovals. **(POINT AND TRACE EACH LITTLE PEAK)**.

### Experimenter-Led Script for the Open-ended Group

**(REFERRING TO THE SAMPLE TOPOGRAPHIC MAP).** Take a look at this topographic map, and describe in as much detail as possible, what you think the terrain it represents looks like. **(ALLOW PARTICIPANT TO LOOK AT MAP AND DESCRIBE.)**

Is there anything else you notice about the map? **(POINT OUT THE PORTION OF THE MAP THE PARTICIPANT HAS NEGLECTED TO DESCRIBE.)**

Okay, let’s focus on the top left corner of the map **(POINT TO BULL’S-EYE PATTERN ON MAP).** Take a look at this section of the topographic map, and describe in as much detail as possible what you think the terrain it represents looks like. **(AFTER PARTICIPANT DESCRIBES.)**

Is there anything you notice about this section of the map? **(IF THE PARTICIPANT NEGLECTED TO TALK ABOUT A REGION, POINT TO THE REGION.)**

Now, let’s focus on the right side of the map **(POINT TO** ***STEEP*** **AND** ***SHALLOW*** **SLOPE REGION ON MAP).**

Take a look at this section of the topographic map, and describe in as much detail as possible what you think the terrain it represents looks like. **(AFTER PARTICIPANT DESCRIBES.)**

Is there anything you notice about this section of the map? **(IF THE PARTICIPANT NEGLECTED TO TALK ABOUT A REGION, POINT TO THE REGION.)**

**(REFERRING TO MAPS 2 AND 3)** Now, we’re going to compare two examples to each other, and get a chance to see how the shapes look in a simulated terrain. Take a moment and compare the maps shown above with their terrains shown below.

First, let’s look at Map 2. Describe in as much detail as possible the topographic map and the terrain it represents. **(AFTER PARTICIPANT DESCRIBES.)**

Is there anything you notice about the map or the terrain? **(IF THE PARTICIPANT NEGLECTED TO TALK ABOUT A REGION, POINT TO THE REGION.)**

Now, let’s look at Map 3. Describe in as much detail as possible the topographic map and the terrain it represents. **(AFTER PARTICIPANT DESCRIBES.)**

Is there anything you notice about the map or the terrain? **(IF THE PARTICIPANT NEGLECTED TO TALK ABOUT A REGION, POINT TO THE REGION.)**

(**REFERRING TO MAPS 4 AND 5)** Now, we’re going to compare two different examples to each other, and get a chance to see how the shapes look in a simulated terrain. Take a moment and compare the maps shown above with their terrains shown below.

First, let’s look at Map 4. Describe in as much detail as possible the topographic map and the terrain it represents. **(AFTER PARTICIPANT DESCRIBES.)**

Is there anything you notice about the map or the terrain? **(IF THE PARTICIPANT NEGLECTED TO TALK ABOUT A REGION, POINT TO THE REGION.)**

Now, let’s look at Map 5. Describe in as much detail as possible the topographic map and the terrain it represents. **(AFTER PARTICIPANT DESCRIBES.)**

Is there anything you notice about the map or the terrain? **(IF THE PARTICIPANT NEGLECTED TO TALK ABOUT A REGION, POINT TO THE REGION.)**

## Abbreviations

ANOVA: Analysis of variance
SOT: Spatial Orientation Test
STEM: Science, technology, engineering, and mathematics
TMA: Topographic Map Assessment
USGS: US Geological Survey
WLT: Water Level Test
